# Predicting IDH subtype of grade 4 astrocytoma and glioblastoma from tumor radiomic patterns extracted from multiparametric magnetic resonance images using a machine learning approach

**DOI:** 10.3389/fonc.2022.879376

**Published:** 2022-09-30

**Authors:** Pashmina Kandalgaonkar, Arpita Sahu, Ann Christy Saju, Akanksha Joshi, Abhishek Mahajan, Meenakshi Thakur, Ayushi Sahay, Sridhar Epari, Shwetabh Sinha, Archya Dasgupta, Abhishek Chatterjee, Prakash Shetty, Aliasgar Moiyadi, Jaiprakash Agarwal, Tejpal Gupta, Jayant S. Goda

**Affiliations:** ^1^ Department of Radiodiagnosis, Tata Memorial Center, Mumbai, India; ^2^ Homi Bhabha National Institute, Mumbai, India; ^3^ Department of Radiation Oncology, Tata Memorial Center, Mumbai, India; ^4^ Department of Pathology, Tata Memorial Center, Mumbai, India; ^5^ Department of Neurosurgery, Tata Memorial Center, Mumbai, India

**Keywords:** glioblastoma, radiomics, molecular subgroups, machine learning, texture feature

## Abstract

**Background and purpose:**

Semantic imaging features have been used for molecular subclassification of high-grade gliomas. Radiomics-based prediction of molecular subgroups has the potential to strategize and individualize therapy. Using MRI texture features, we propose to distinguish between IDH wild type and IDH mutant type high grade gliomas.

**Methods:**

Between 2013 and 2020, 100 patients were retrospectively analyzed for the radiomics study. Immunohistochemistry of the pathological specimen was used to initially identify patients for the IDH mutant/wild phenotype and was then confirmed by Sanger’s sequencing. Image texture analysis was performed on contrast-enhanced T1 (T1C) and T2 weighted (T2W) MR images. Manual segmentation was performed on MR image slices followed by single-slice multiple sampling image augmentation. Both whole tumor multislice segmentation and single-slice multiple sampling approaches were used to arrive at the best model. Radiomic features were extracted, which included first-order features, second-order (GLCM—Grey level co-occurrence matrix), and shape features. Feature enrichment was done using LASSO (Least Absolute Shrinkage and Selection Operator) regression, followed by radiomic classification using Support Vector Machine (SVM) and a 10-fold cross-validation strategy for model development. The area under the Receiver Operator Characteristic (ROC) curve and predictive accuracy were used as diagnostic metrics to evaluate the model to classify IDH mutant and wild-type subgroups.

**Results:**

Multislice analysis resulted in a better model compared to the single-slice multiple-sampling approach. A total of 164 MR-based texture features were extracted, out of which LASSO regression identified 14 distinctive GLCM features for the endpoint, which were used for further model development. The best model was achieved by using combined T1C and T2W MR images using a Quadratic Support Vector Machine Classifier and a 10-fold internal cross-validation approach, which demonstrated a predictive accuracy of 89% with an AUC of 0.89 for each IDH mutant and IDH wild subgroup.

**Conclusion:**

A machine learning classifier of radiomic features extracted from multiparametric MRI images (T1C and T2w) provides important diagnostic information for the non-invasive prediction of the IDH mutant or wild-type phenotype of high-grade gliomas and may have potential use in either escalating or de-escalating adjuvant therapy for gliomas or for using targeted agents in the future.

## Introduction

High-grade gliomas, especially grade 4 astrocytomas and glioblastomas, are not only the most common primary malignant brain tumors in the adult population but are also associated with intrinsic heterogeneity and invasive properties and are clinically associated with high morbidity and lethality (1). With a better understanding of biology and the advent of newer molecular techniques, researchers have been able to develop unique biomarkers that could predict treatment response and predict these tumors with a high degree of accuracy, paving the way for a more personalized treatment approach. The two molecular biomarkers of significant interest that have translated into clinical practice are Isocitrate Dehydrogenase (IDH) and MGMT (O (2)-methylguanine-DNA methyltransferase), both of which are responsible for epigenetic alterations in glioblastomas. The evaluation of these biomarkers has now become the norm in tailoring therapy and disease prediction.

Glioblastomas, although previously categorized under grade 4 gliomas, are now considered biologically and molecularly distinct entities, namely, glioblastoma IDH-wildtype and IDH-mutant grade 4 astrocytoma, based on ‘the present’ World Health Organization classification of brain tumors. IDH mutations are identified in approximately 5%–13% of glioblastomas and are associated with a significantly better prognosis, particularly when resection includes the non-enhancing tumor component, which is traditionally left unresected (3). Therefore, it is essential to distinguish the IDH mutation status for planning the most appropriate management strategies, as IDH-mutated tumors have more prolonged overall survival and a higher chance of responding to chemotherapy or radiotherapy (4, 5).

Currently, IDH mutation status is assessed by immunohistochemistry (IHC) or DNA sequencing techniques of the tumor specimen, which is invasive, and given the morphological heterogeneity and invasiveness of high-grade gliomas, the full extent of intratumoral phenotypic/genotypic heterogeneity may not be represented in the tumor specimen. Additionally, the widespread use of these biomarkers remains a challenge due to either a lack of expertise or cost issues associated with their testing. For these reasons, accurate preoperative assessment of the IDH mutation from radiological images is important for prognostic evaluation and optimizing therapy for high-grade gliomas (which in our study are grade 4 astrocytoma, IDH-mutant, and glioblastoma, IDH-wildtype).

Studies have demonstrated that certain quantitative image features, like texture features, can be used to predict both IDH mutations on preoperative imaging of gliomas (6). Tumor radiomics based on texture analysis of MR images represent a quantitative approach in which several individual imaging features that are not easily perceived by the unaided eye are processed using advanced algorithms to reveal measurable indices. Given the inherent tumor heterogeneity in histopathological tissues and the universal availability of MRI, we expected the use of machine learning classifiers of the tumor texture features extracted from multiparametric magnetic resonance imaging (MRI) in a large cohort of GBM patients to subclassify them based on the IDH status as confirmed by immunohistochemistry and/or gene sequencing as the gold standard. The study aimed to explore the accuracy of MR-based tumor radiomics and develop a robust model using a machine learning approach to classify GBM into two distinct molecular subgroups of IDH wild and IDH mutant types in a fairly large cohort of patients.

In this retrospective single-center study, we developed a simple radiomics model using a Support Vector Machine algorithm, based on a minimal set of tumor features obtained using a single and multislice tumor segmentation approach on multiparametric MRI sequences for pretreatment prediction of IDH1 status in high-grade glioma patients.

## Materials and methods

### Patient population

The study was initiated at a tertiary cancer care center through an institutional intramural grant (Grant no. TRAC/1016/1710/001) after obtaining due approval from the Institutional Ethics Committee (IEC). All histologically confirmed high-grade glioma patients, patients who had complete clinical and pretreatment imaging data in Digital Imaging and Communications in Medicine (DICOM) format, and patients whose IDH status was determined by immunohistochemistry and/or Sanger sequencing were included in the study for radiomic feature extraction, classification, and building the model for sub-classifying the high-grade gliomas based on their IDH status.

### Molecular subtyping

IDH mutant or wild phenotype was classified by initial screening using immunohistochemistry (IHC) of the paraffin-embedded tissue followed by DNA sequencing in cases where IHC results were equivocal as per the institutional protocol. The IDH R132H mutation was tested by IHC for all the glial tumors. The antibody used for IDH immunohistochemistry was mouse monoclonal anti-IDH1R132H, clone H09 from Dianova GMBH (Hamburg, Germany). Tumors that stained for IDH antibody were considered positive for IDH mutations, while tumors that did not stain for IDH were subjected to Sanger sequencing, considered the gold standard for detecting IDH mutations. Sanger sequencing for IDH1R132 and IDH2R172 loci was performed by PCR using specific primers from Sigma-Aldrich. On sequencing, other alterations besides the commonest R132H were identified. If sequencing was negative, an absence of IDH mutation was confirmed, and such tumors were deemed IDH wild-type GBM. If IHC was negative and sequencing was positive, such tumors were considered IDH mutant (2).

### Radiomics pipeline

**Figure 1:**
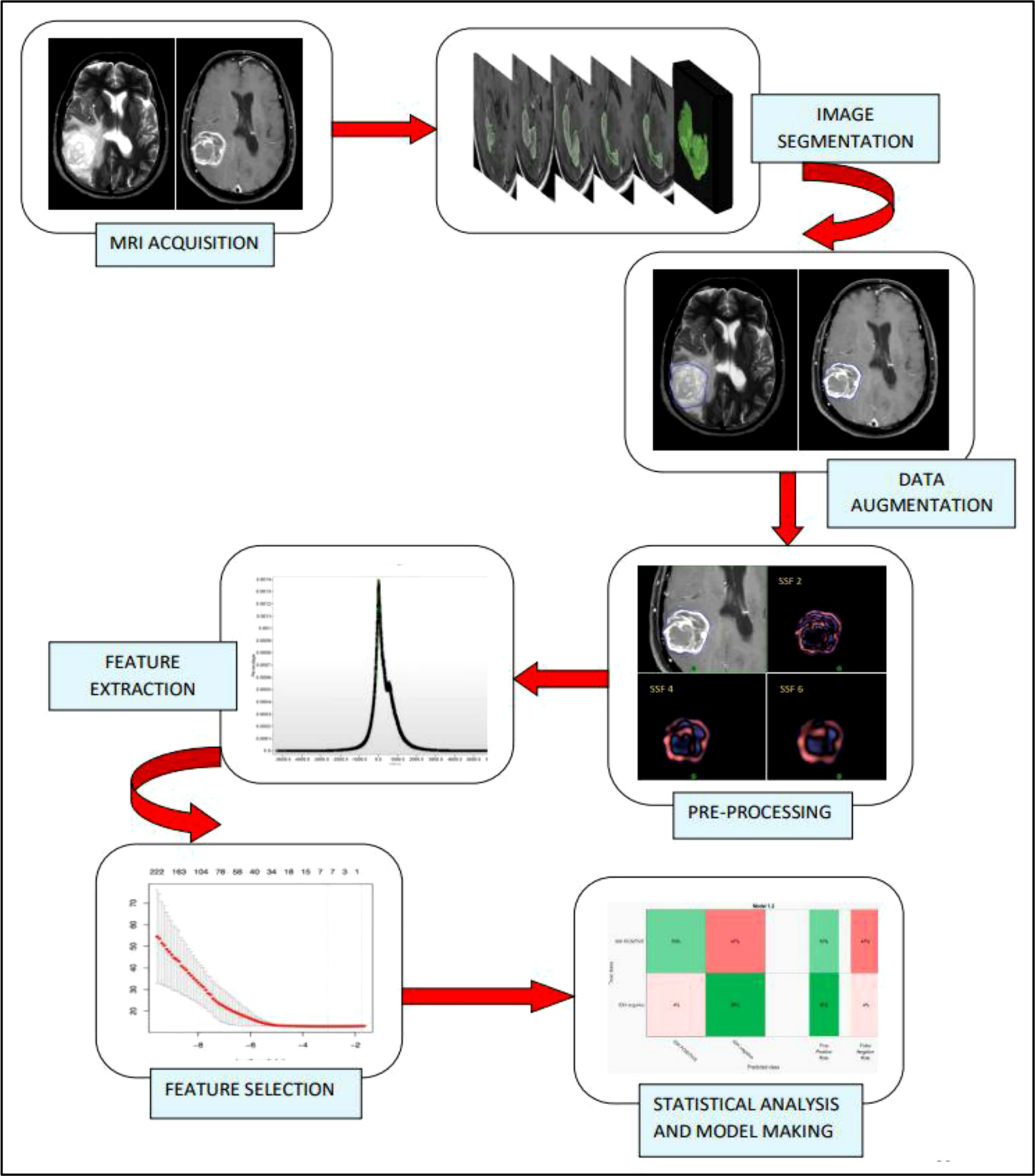
Radiomics study flow. The radiomic workflow involves MR brain imaging and data acquisition, followed by slice by slice image segmentation, data augmentation by single slice multiple sampling technique, Image pre-processing by spatial scale filters which involve the use of LoG (Laplacian of Gaussian) bandpass filter, extraction of first order, and second-order features from the texture analysis software, feature selection using LASSO regression and statistical analysis and model development using Support Vector Machine (SVM) and a 10-fold cross-validation strategy.

A visualization of the steps in the radiomics workflow is depicted in **Figure 1**. Initially, the brain tumor images were acquired from two different MRI machines (1.5 Tesla Philips™ and 3 Tesla General Electric™). The DICOM compatible images were imported into the TexRad software™ and reconstructed. The reconstructed images were preprocessed using spatial scaled filters (SSFs) to reduce the background noise and increase the sharpness of the tumor edges. The preprocessed images were used to contour the region of interest (ROI). The segmented images were augmented to increase the number of image data sets. Shape, first order (or histogram), and second order texture (GLCM) features were then extracted from the region of interest. The extracted features were then scaled down using the LASSO regression method. Finally, the data analysis step involved building a model from the selected radiomic features to predict the endpoint of interest (IDH wild vs. IDH mutant high-grade glioma).

### Image acquisition protocol

Magnetic resonance imaging sequences of 100 patients were obtained at our institution using Philips Ingenia 1.5T and GE Signa 3T MRI with a pre-fixed standard scanning protocol for brain tumor imaging. Axial T1 contrast (T1C) and T2W images were obtained from the vertex to the skull base, encompassing the whole brain, where the primary tumor is visible in its entirety. These sequences were archived in the institutional Picture Archival and Communication System (PACS) and transferred to the radiomics (texture) analysis system (TexRAD™). The radiological features on the T2W and contrast-enhanced T1W MR images were evaluated and discerned by an experienced neuro-radiologist, and the texture features were extracted on the TexRad™ console.

### MR image preprocessing, segmentation (ROI generation), and augmentation

Magnetic Resonance Imaging of the brain was acquired on two different MRI machines (1.5 Tesla Phillips™ and 3 Tesla General Electric™). The acquisition details of the MR images for the brain imaging protocol for both machines have been explicitly described in **Table 1**. The resultant imaging protocol will result in some imaging heterogeneity. Therefore, before segmentation and ROI delineation, image preprocessing was performed using the Laplacian of Gaussian (LOG) bandpass filters to remove the background noise (Gaussian filter) and enhance the tumor edges (Laplacian filter). This allowed for the extraction of specific structures corresponding to the filter width. Spatial scale Filters (SSF) used filtration values of 0, 2 mm, 3 mm, 4 mm, 5 mm, and 6 mm in width (radius), representing the increasingly coarser level of texture scales for first-order statistics. The use of a filtration algorithm before radiomic feature extraction helps in nullifying some of the effects of heterogeneous acquisition protocols and improves the robustness of the feature selection by removing the features affected by MR noise and imaging heterogeneity.

**Table 1:** MR image acquisition protocol.

MRI Machine	Sequences	FOV (cm)	Matrix	NEX	Slice thickness (mm): Slice gap (mm)
**GE Signa 3T**	Axial T2	24	320 × 224	1	5:1.5
	Axial T1 + C	24	320 × 190	1	5:1.5
**Philips Ingenia 1.5T**	Axial T2	23 (AP) 18.5 (RL)	448 × 304	2	5:1
	Axial T1 + C	23 (AP) 18.5 (RL)	232 × 104	2	5:1

FOV, Field-of-view; NEX, Number of excitations; AP, Anteroposterior; RL, Right left.

Tumor segmentation and region of interest (ROI) delineation were performed manually with the freehand drawing function (polygon tool) of the software. The ROI contours and segmentation were separately verified by a neuro-oncologist with 10 years of experience and a neuroradiologist with 10 years of experience. The segmentation was verified by them individually, and any discrepancy was resolved by a consensus. For analysis, the final contours as verified by the neuroradiologist were considered. Two types of segmentation techniques were used, i.e., whole tumor segmentation (volumetric) as well as single slice with multiple sampling segmentation methods, which in turn were used for data augmentation as described in prior literature (7, 8). A total of 831 Axial T1C and 831 T2 image datasets were obtained for analysis from the study population.

### MR texture analysis

**Figure 2:**
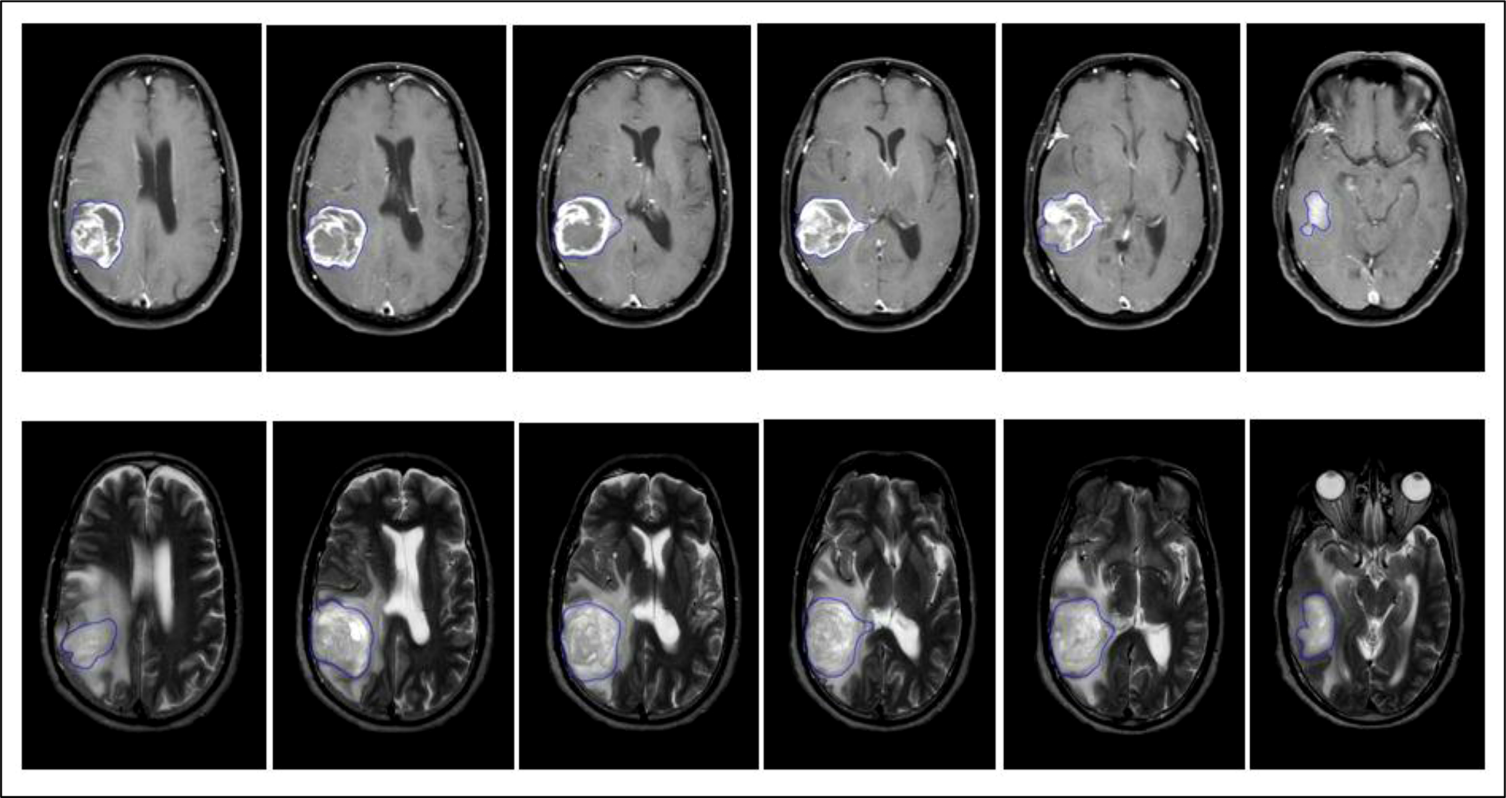
Representative multi-slice region of interest (ROI) of an IDH wild-type GBM done on axial T1 + C and T2 MR Images using slice-by-slice image segmentation.

**Figure 3:**
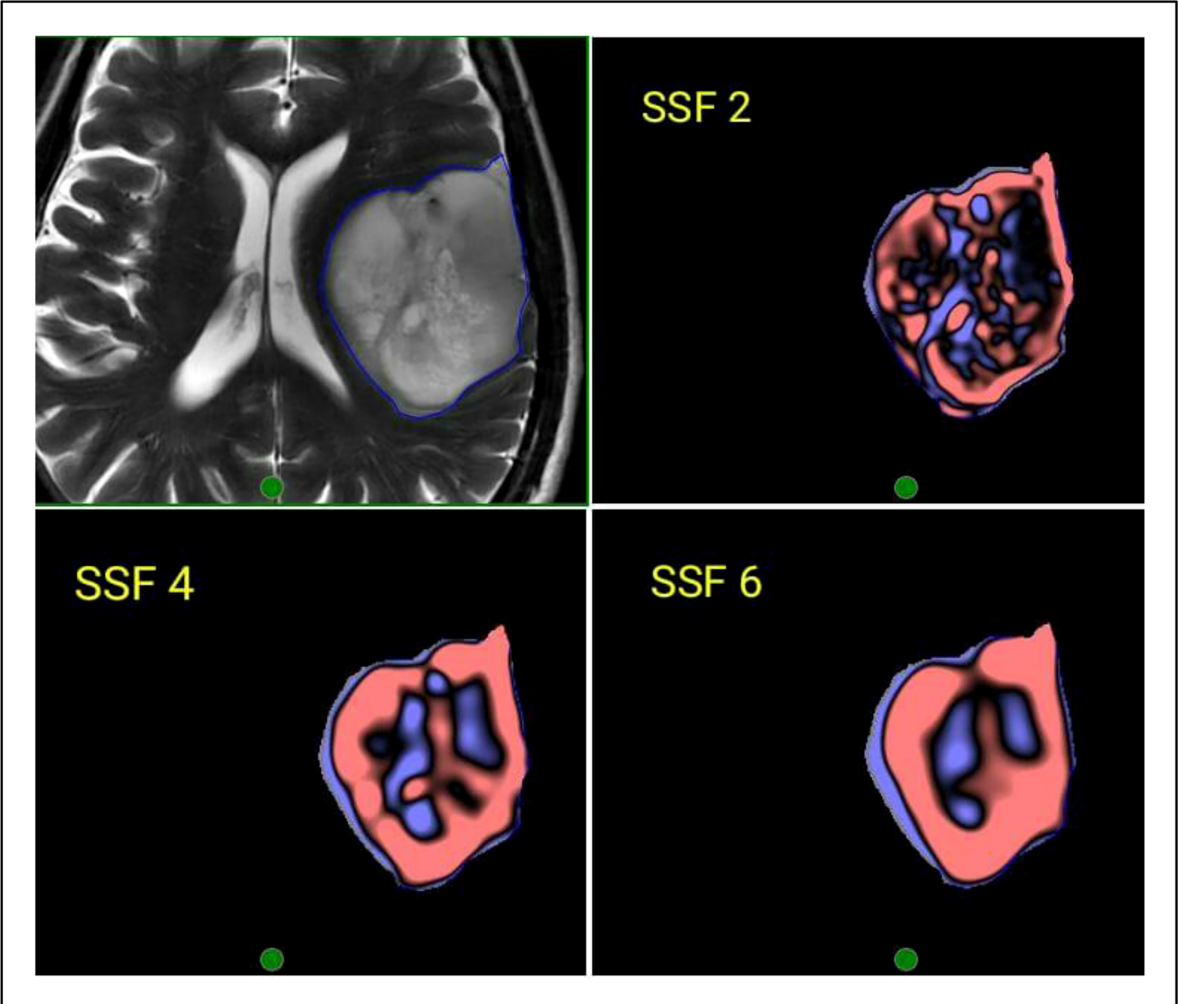
Representative image of the region of interest (ROI) contoured on a T2W MRI and corresponding filtered images using Laplacian of Gaussian (LOG) bandpass filtration algorithm showing SSF-2 (fine texture), SSF 4 (Medium texture), and SSF 6 (Coarse texture).

The radiomic features were extracted from the segmented images using proprietary texture analysis research software (TexRAD™ Research Version 3.10, TexRAD Ltd, Cambridge, UK), and the machine learning algorithm (SVM) developed a predictive model for molecular sub-classification of high-grade gliomas and was blinded to molecular diagnosis. Eighty-two radiomic features were extracted separately for T1W + C and T2W images using the TexRAD tool, which included 36 first-order features at various SSFs (0, 2, 3, 4, and 6) (**Figure 3**). Second-order features such as Gray Level Co-occurrence Matrices (GLCM) and topographic features were extracted without applying filters. Twenty GLCM features each for pixel pairs spaced 1 pixel (GLCM1) and 4 pixels apart (GLCM4) respectively, and 6 Shape features **(** **Figure 2** **).** The details of all the texture features are provided in **Table 2**.

**Table 2:** Demographic, tumor and treatment profile of grade 4 IDH mutant astrocytoma and IDH wild type glioblastoma.

			Overall(Total N = 100)		IDH Wild (N = 83)	IDH Mutant (N = 17)	p-value
**BASELINE CHARACTERISTICS**							
**AGE**							
** Median age**			52 years		54 years	34 years	< 0.001
** Range**			19–71 years		19–71 years	23–68 years	
** IQR**			38–59 years		46–59 years	27–43 years	
**GENDER**							
** Male**			70		58 (69.9%)	12 (70.6%)	0.954
** Female**			30		25 (30.1%)	5 (29.4%)	
**CENTRICITY**							
** Unicentric**			94		78 (94%)	16 (94%)	0.982
** Multicentric**			6		5 (6%)	1 (6%)
**LATERALITY**							
** Right**			37		33 (39.8%)	4 (23.5%)	0.450
** Left**			53		42 (50.6%)	11 (64.%)
** Central/Bilateral**			10		8 (9.6%)	2 (11.8%)
**LOCATION**							
** Cerebellum**			2		2 (2.4%)	0 (0%)	0.479
** Frontal**			31		20 (24.1%)	11 (64.7%)
** Insular**			2		2 (2.4%)	0 (0%)
** More than two**			32		29 (34.9%)	3 (17.6%)
** Occipital**			2		2 (2.4%)	0 (0%)
** Parietal**			17		15 (18.1%)	2 (11.8%)
** Temporal**			14		13 (15.7%)	1 (5.9%)
**HISTOPATHOLOGY**							
**MGMT**							
** Unmethylated**				36	32 (48.5%)	4 (33.3%)	0.333
** Methylated**				42	34 (51.5%)	8 (66.7%)
** Unknown**				22			
**ATRX**							
** Retained**				73	71 (88.8%)	3 (17.6%)	< 0.001
** Lost**				15	5 (6.3%)	10 (58.8%)
** Non-contributory**				8	4 (5.0%)	4 (23.5%)
** Unknown**				3
**Overall**
** P53**							
** Negative**				2	2 (2.4%)	0 (0%)	0.518
** Positive**				98	81 (97.6%)	17 (100%)
** Median Mib 1 index (%)**				17.5 (IQR 4%–55.5%)	17.5 (IQR 13.5–22.5)	17.5 (IQR 8–23.75)	0.188
**TREATMENT DETAILS**							
**EXTENT OF SURGERY (n = 99)**							
** Gross total resection**			34		31 (37.8%)	3 (17.6%)	0.271
** Near-total resection**			26		20 (24.4%)	6 (35.3%)
** Subtotal resection**			39		31 (37.8%)	8 (47.1%)
** RADIOTHERAPY**							
** RT received**		Yes	88		72 (86.7%)	16 (94.1%)	0.451
		No	12		11 (13.3%)	1 (5.9%)
** Median RT dose**			59.4 Gy, Range (56.5 Gy to 59.4 Gy)		59.4 Gy, Range (56.5 Gy to 59.4 Gy)	59.4 Gy, Range (56.7 Gy to 59.4 Gy)	0.781
** Median RT fractions**			33 (IQR 30 to 33 fractions)		33 (IQR 30 to 33 fractions)	33 (IQR 31 to 33 fractions)	0.451
**ADJUVANT TMZ (Temozolomide)**							
**Adj. TMZ Received**	Yes		74		60 (72.3%)	14 (82.4%)	0.389
	No		26		23 (27.7%)	3 (17.6%)
**Median cycles of adjuvant TMZ**			6 (IQR 4.25–11)		6 (IQR 4–6.50)	11 (IQR 6–12)	0.038

IQR, Inter quartile range; TMZ, Temozolomide; RT, Radiation Therapy; ATRX, Alpha-Thalassemia/Mental Retardation Syndrome, X-Linked; MGMT, O^6^-Methylguanine-DNA Methyltransferase.

### Radiomic feature selection

The least absolute shrinkage and selection operator (LASSO) logistic regression algorithm was used for reducing the excessive dimensionality of data and selecting the most significant features in the training data set. Radiomic features with non-zero coefficients were selected from the training data. The analysis was performed using R™ software version 3.6.3, Vienna, Austria, and R Studio™ version 1.2.5033, Boston, USA using the “glmnet” package.

### Radiomic feature classification and modeling

The features selected by LASSO were used as a training set for model development. A Support Vector Machine (SVM) classifier with a 10-fold cross-validation strategy was used in the prediction of the two main molecular subgroups. The performance of the model was assessed using the Area Under Curve (AUC). Multiple models were sequentially evaluated by the system using a combination of selected texture features to arrive at the best model. The SVM analysis was conducted with MATLAB™ version 9.0 (R2016a), The MathWorks, Inc., Natick, MA, USA. Standardization (z-score normalization) was done on the extracted features before SVM analysis as the predictors were of different scales.

### Statistical analysis

Quantitative variables were expressed as mean and/or median. The Student t-test for independent samples was used for the comparison of two different groups. In the case of variables that were not distributed normally, the Mann–Whitney rank sum test was used. The diagnostic accuracy for IDH genotype prediction by textural features was evaluated by analyses of receiver-operating characteristic (ROC) curves using immunohistochemistry/gene sequencing results as the gold standard. The area under the ROC curve (AUC) was evaluated to assess the performance of the developed model. The diagnostic metrics used to assess the model were the AUC, sensitivity, specificity, and overall accuracy as reported in various literature studies investigating Machine Learning-Based Radiomics Signatures for different types of cancers (9–11).

### Radiomics quality assurance score and the image biomarker standardization initiative

Imaging data for extracting radiomic features have been used as a tool for testing medical hypotheses. However, the radiomic features extracted from the image data had high dimensionality, requiring complex models to predict or correlate with the endpoints of interest. This limits its usage for only research purposes without real-world application in the clinics and guides the clinical decision-making process, resulting in a huge translational gap. Therefore, Lambin et al. developed a standardized radiomic quality assurance score (RQS) for evaluating the performance, reproducibility, and/or clinical utility of radiomic biomarkers. The RQS is a reporting system of metrics used to validate the robustness of radiomic studies (12). The RQS comprises 16 components, as represented in **Supplementary Table 1**.

Apart from the RQS, our study tried to adhere to the Image Biomarker Standardization Initiative (IBSI) guidelines which were initiated to address the challenges in utilizing radiomics as an image-based biomarker (13) For this study, we evaluated all the processing steps from image processing, segmentation, and ROI delineation to the computation of radiomic features were evaluated in this study **(** **Supplementary Table 2** **).**

## Results

### Baseline demographics and tumor and treatment characteristics of the study cohort

One hundred and thirty-three patients with a histological diagnosis of high-grade gliomas (CNS WHO grade 4 of adult type diffuse gliomas) were screened for the radiomic study. Based on the inclusion criteria, only a hundred patients were eligible for the study. Seventeen patients had IDH mutations and 83 patients had IDH wild-type glioblastoma. The median age of patients at presentation was 52 years (a range of 19 to 71 years) and the majority of them were males (70%), The demographic details of the study population are presented in detail in **Table 2**. All but one patient underwent maximal safe resection of the tumor, whereas one patient underwent only biopsy, followed by risk-based adjuvant therapy incorporating both radiotherapy and chemotherapy as deemed appropriate after discussion in a joint multidisciplinary clinic **Table 3**.

**Table 3:** Radiomic features extracted.

Texture Features Used	
**1^st^ Order Features**	Mean Standard Deviation Mean of Positive pixels Entropy Skewness Kurtosis
**GLCM features**	Autocorrelation Cluster prominence Cluster shade Cluster tendency Contrast Correlation Dissimilarity Homogeneity Joint average Joint energy Joint entropy Idm (inverse difference moment) Diffentropy Diffvariance Idmn (inverse difference moment normalized) Idn (inverse difference normalized) Inverse variance Sum entropy Sum squares Join tmax
**Shape Features**	Perimeter Area Elongation Sphericity Long axis Short axis

### Molecular subgrouping

Of the 100 patients who were studied, IHC for **IDH1R132H** was done on all the cases. IDH1/2 sequencing was performed on cases that were deemed negative on IHC for **IDH1R132H** but showed loss of expression for ATRX. The cases which were negative for **IDH1R132H** on IHC and showed retained expression of ATRX were taken as IDH wild type (14). A total of 13 patients (13%) were positive for **IDH1R132H** on IHC. Eighty-seven patients (87%) were negative for IDH1R132H on immuno-histochemistry. Among the 87 patients, six showed loss of expression of ATRX and underwent Sanger sequencing for confirmation of IDH status. Of these six patients, four showed IDH mutations on Sanger sequencing: two patients were positive for **IDH1R132C** only, while one patient had an **IDH1R132L** mutation and another patient showed an **IDH1R132H** mutation. Two of the six patients showed no point mutation for IDH1 or IDH2 and were considered IDH-wild type. Therefore, of the 100 patients, 17 patients were considered IDH mutant subtype, while 83 patients were IDH wildtype.

### Performance of the binary classification model

Out of a total of 82 texture features each in T1W + C and T2W images, LASSO regression for feature selection elucidated seven discriminant features for T1W + C images and seven discriminant features for T2W images, which were used for further model development.

**Figure 4:**
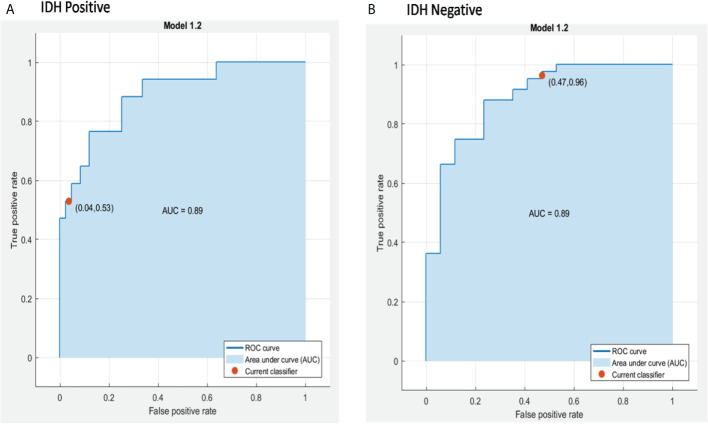
ROC curves of the best model for prediction of the two molecular subgroups using combined multi-slice T1 + C and T2w GLCM features using Quadratic SVM, **(A)** IDH positive and **(B)** IDH negative.

**Figure 5:**
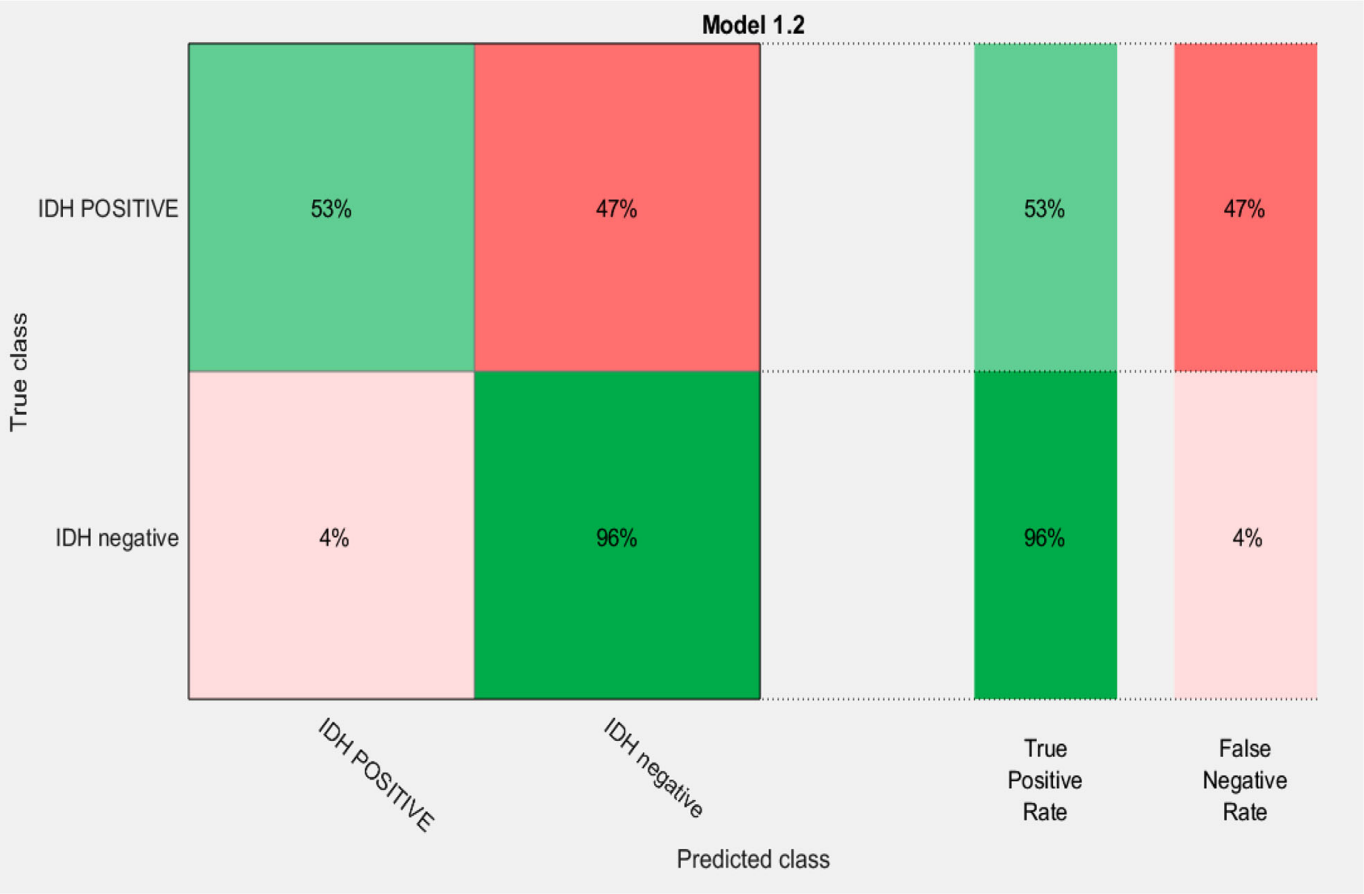
Confusion matrix of the best model for prediction of the two molecular subgroups using GLCM features of combined multi-slice T1 + C and T2w images using Quadratic SVM.

A combination of LASSO selected first order texture features, second order (GLCM) features, and topographic features were used to create different models using both T1W + C and T2W images in an attempt to arrive at the best SVM model **Table 5**. Among various models evaluated, a combination of 14 GLCM features from combined T1W + C and T2W images resulted in the best classifier, as depicted in **Table 4**. The model based on a Combined Multi-slice Texture Analysis of T1 + C and T2 weighted MR imaging using a Quadratic Support Vector Machine Classifier and a 10-fold internal cross-validation approach, resulted in the best performance in predicting the molecular subtypes with a predictive accuracy of 89% and a Receiver Operator Characteristic (ROC) analysis demonstrating an AUC of 0.89 for each IDH positive and IDH negative subtype **(** **Figure 4** **).** Of the 83 IDH negative cases, 80 tumors were true positive while three tumors were false negative, resulting in a very high sensitivity of 96%, but at the same time, the model specificity was 52.9%. This low specificity is due to the unbalanced classification of IDH subtypes. Similarly, for 17 IDH positive cases, nine tumors were true positives while eight tumors were false negatives, resulting in a sensitivity of only 53% but a high specificity of 96.4% as depicted in the confusion matrix **(** **Figure 5**), **Table 6**.

**Table 4:** A combination of LASSO selected features that resulted in the best classification model.

T1W + C TEXTURE FEATURES (N = 7)	T2W TEXTURE FEATURES (N = 7)
KURTOSIS_0_T1C	MEAN_0_T2
ENTROPY_2_T1C	MPP_0_T2
KURTOSIS_2_T1C	KURTOSIS_0_T2
MEAN_5_T1C	MEAN_4_T2
KURTOSIS_5_T1C	GLCM1_clusterShade_T2
SKEWNESS_6_T1C	GLCM1_idn_T2
GLCM4_correlation_T1C	GLCM1_sumEntropy_T2

**GLCM 1**, GLCM features of pair of pixels which are 1 pixel apart; **GLCM 4**, GLCM features of a pair of pixels which are 4 pixels apart; **T1C**, Contrast-enhanced T1 weighted images; **idn**, inverse difference normalized.

**Table 5:** Showing the molecular classification (IDH mutant and IDH wild type) of grade-IV GBM modeled by using Support Vector Machine as the radiomics classifier on MRI-based sequences.

ImageSingle slice v/s Multi slice	MRI sequence	IDHClassification	Radiomics classifier	Diagnostic Metrics		Validation Process	
				AUC	Accuracy	10-fold internal cross-validation	Hold Validation
Single slice analysis	T1C	IDH –VE (694)	Linear SVM	0.91	89.8%	YES	NO
		IDH +VE (137)		0.91			
						
	T2W	IDH –VE (689)	Cubic SVM	0.84	86.9%	YES	NO
		IDH +VE (149)		0.84			

Multi-slice analysis	T1C	IDH –VE (83)	Linear SVM	0.87	87%	YES	NO
		IDH +VE (17)		0.87			
						
	T2W	IDH –VE (83)	Quadratic SVM	0.80	91%	YES	NO
		IDH +VE (17)		0.80			
						
	T1C + T2W	IDH –VE (83)	Quadratic SVM	0.89	89%	YES	NO
		IDH +VE (17)		0.89			
						
	T1C + T2W	IDH –VE (83)	Cubic SVM	0.81	90%	NO	YES (90:10)
		IDH +VE (17)		0.81			

AUC, Area under the curve; SVM, Support Vector Machine.

**Table 6:** Performance of best classification model.

Diagnostic metrics	IDH −VE (n = 83)	IDH +VE (n = 17)
**AUC**	**0.89**	**0.89**
**TP**	80	9
**TN**	9	80
**FP**	8	3
**FN**	3	8
**Sensitivity**	96%	53%
**Specificity**	52.9%	96.4%
**FNR**	4%	47%
**PPV**	90.9%	75%
**NPV**	75%	90.9%
**Overall Accuracy**	**89%**	

AUC, Area under the curve;

TP, True positive; TN, True negative; FP, False positive; FN, False negative; FNR, False negative rate; PPV, Positive predictive value; NPV, Negative predictive value.

## Discussion

We developed a Support Vector Machine (SVM) based classification model with satisfactory performance to probe the genomic profile (IDH mutant vs. IDH wild type) of grade 4 adult diffuse gliomas, based on MR image phenotypes. The SVM classifier had an overall accuracy of 89% for predicting IDH wild-type tumors from IDH mutants. Our results suggest the use of multiparametric MR radiomics along with machine-learning models to classify the molecular subtype of grade 4 adult type diffuse gliomas consistent with the new 2021 WHO classification. By employing a specific ML classifier, several clinical applications for the detection of IDH status in high-grade gliomas can be achieved with or without histopathology of the tumor specimen.

IDH mutations are considered to be an early event in gliomagenesis and are one of the most critical genetic biomarkers for high-grade gliomas having prognostic implications (improved survival with IDH mutant than wild-type glioblastomas {31 months vs 15 months}) (15). Additionally, IDH1 mutation is sufficient to establish the glioma hypermethylator phenotype, which is a powerful determinant of tumor pathogenicity (16). Therefore, having a preoperative assessment of IDH gene mutation status in glioma may help in optimizing glioma therapeutics. While immunohistochemistry is considered a routine screening method for detecting IDH mutations in the majority of cases, Sanger sequencing is considered to be a confirmatory test for identifying IDH mutations. However, high-grade gliomas, especially glioblastomas, show marked intratumoral heterogeneity in IDH status. Pathological tissue biopsies from the different parts of tumors may yield varied results regarding the IDH status as these high-grade gliomas are considered to be heterogeneous. Therefore, a non-invasive method like magnetic resonance imaging could be put to effective use for objectively quantifying structural heterogeneity within the tumor using image-based radiomic analysis. Radiomics is a novel approach for the high-throughput extraction of quantitative image features from a specified ROI (17). These quantitative features (radiomic features) have been successfully used to develop models using sophisticated machine learning algorithms for identifying image biomarkers with the capability to predict the genotype of a tumor (18). Published studies have leveraged machine learning classifiers to develop radiomic signatures to predict IDH mutation status in gliomas (11, 19, 20). Within the framework of radiomics, tumor texture features as extracted from MR images of brain tumors are predefined and quantitative features are derived by computational methods that describe the spatial variations in the intensity of the images along the entire cross-section of the tumor that is beyond visual perception. These features have the potential to yield additional information not only about the tumor biology but also about the genomic profile. Thus, they allow the prediction of the IDH genotype in glioma patients with a high degree of accuracy (21). The present study was done to investigate the feasibility of using machine learning-based radiomic signatures to predict the IDH subtype in high-grade gliomas in a high throughput setting.

Radiomics-based machine learning tools or deep learning tools have been used for subclassifying various grades of gliomas into IDH wild-type or mutant-type entities (22). However, the literature on this subject is quite sparse **(** **Table 7** **)**. A Taiwanese group used radiomic features consisting of morphological, intensity, and textural features to develop a prediction model for IDH mutation (26) and textural features yielded the best accuracy of 85%. Going further, the group used the same set of patients to interpret the status of IDH status in glioblastomas from transformed magnetic resonance imaging patterns (26). By ranklet transformation of collected images from 39 patients (32 IDH wild and seven IDH mutant cases), three feature sets were extracted, with each feature set having 14 GLCM textural features. They achieved an accuracy of 90%, a sensitivity of 57%, and a specificity of 97%. In contrast to the Taiwanese group, our study used both axial T2 and axial post-contrast T1 + C images, and unlike the largest single slice that was used in this study, we incorporated tumor contours on each axial slice of both the sequences wherever the tumor was present. This took into account the heterogeneity present within the entire tumor volume, which has an advantage over core biopsy methods, which target only a limited section of the tumor for histopathology.

**Table 7:** Literature review of studies using radiomics and or semantic features for glioblastoma molecular subgroup classification using various diagnostic metrics.

No.	Author(year)No. of patients	MRI sequences	Model used for subgroup classification		AUC	Sensitivity	Specificity	PPV
3	Hsieh et al. (23) (2020), (n = 39)	Feature-based with use of ranklet transformation on axial T1 + C MR images	KNN and SVM	Test Cohort	–	0.57	–	–
	Pasquini et al. (24) (2021), (n = 100)	Featureless radiomics on MPRAGE, FLAIR, T1W, T2W, DWI with ADC, PWI) with DSC sequence	4 block 2D-CNN architecture	Training and test (80:20) set.	0.86 ± 0.05, the highest achieved using rCBV maps	0.76 ± 0.05	–	–
2	Calabrese et al. (25) (2020), (n = 199)	Fully automated deep learning-based tumor segmentations using T1W, T2W, T2W/FLAIR, DWI, SWI, HARDI fractional anisotropy (HARDI FA), ASL, and T1C.	Automated dCNN segmentation	10-fold stratified shuffle split cross-validation strategy with a train/test split of 60:40	0.95 ± 0.03	0.93 ± 0.08	–	–
4	Pashmina et al. (Present study) (n = 100)	Feature-based radiomics using axial T1 + C and T2W MR images	LASSO regression and SVM	10-fold internal cross-validation	0.89	0.96 for IDH wild, 0.80 for IDH mutant	0.53 for IDH wild, 0.03 for IDH mutant	0.91 for IDH wild, 0.75 for IDH mutant

SVM, Support Vector Machine; LASSO, Least absolute shrinkage and selection operator; CNN, Convolutional neural network; IDH, Isocitrate dehydrogenase; ADC, apparent Diffusion Coefficient; DSC, Dynamic Susceptibility Contrast; PWI, perfusion-weighted images.

Comparative models studying the predictive abilities of radiomic features have been rarely performed in the literature. A multicentric study compared various machine learning classifiers to predict the genetics of GBM on different MRI sequences. This study was done on 156 adult patients with a pathologic diagnosis of GBM. Radiomic features were extracted using various extraction tools like NET, CET, and NEC with a custom version of Pyradiomics and selected through the Boruta algorithm. The investigators used various radiomic classifiers like AdaBoost (AB), Extreme Gradient Boosting (xGB), Gradient Boosting (GB), Decision Tree (DT), and Random Forest (RF), Logistic Regressor (LR), two stacking classifiers (ST, ST_ABC), and K Neighbors (KN). It is used to classify IDH mutants from the IDH wild subtype of GBM. Based on the results, the AB classifier performed the best, with a reported accuracy for classifying the IDH phenotype. (overall accuracy of 89% and ROC-AUC of 87.7%) (27). The SVM classifier we used to predict the IDH subtype performed relatively well (ROC-AUC of 89% and overall accuracy of 89%, similar to the above study) (27).

Isocitrate dehydrogenase (IDH) mutations are quite common in low-grade gliomas, unlike in higher grade gliomas. Machine learning-based radiomic feature modeling has been tried in various grades of gliomas (28). Sakai et al. in a heterogeneous cohort of gliomas [n = 100 (grade-I I = 11; grade-3 = 8 and grade IV: 81)] used MRI-based radiomic features to predict IDH1 Mutation Status in Gliomas using a gradient tree boost machine learning classifier. The best performance was seen with a DWI-trained XG Boost model, which achieved ROC with an Area Under the Curve (AUC) of 0.97, an accuracy of 0.90 on the test set. They used the same machine learning classifier (XG boost) on the FLAIR-MR images used as a test set and achieved a ROC with an AUC of 0.95 and an accuracy of 0.90. Their results showed that the model that was trained on combined FLAIR-DWI radiomic features did not provide an increment in terms of accuracy. Using multiparametric radiomic features derived from preoperative MRI can predict IDH1 mutation status with approximately 90% accuracy (28).

Although a single institutional study, the radiomic analysis and model development were done on a relatively small sample size. In our study, we used two approaches to analyze the texture data: a volumetric approach and a single slice multiple sampling approach. Analysis was done using a Support Vector Machine classifier based on features selected by LASSO regression, which selected the best of all the features. Support Vector Machine utilizes the concept of a hyperplane, which is a plane that has the maximum margin, and considers the furthest of the points falling on either side of the hyperplane and is less vulnerable to overfitting as compared to other simple classifiers like logistic regression. Moreover, outliers have less impact on the SVM as opposed to other machine learning algorithms, especially when in higher dimensional data. Various classifier models were used and validation was done using 10-fold internal cross-validation as well as hold-out validation at ratios of 9:1, 8:2, and 7:3, and the latter yielded suboptimal results due to a lack of adequate sample size. The texture features analyzed included first-order and GLCM features. To overcome the limitation of the small sample size, an augmentation strategy called the single slice multiple sampling approach was evaluated. This approach enabled us to reduce the potential overfitting of data, which is known to happen in machine learning approaches, and this approach also yielded appreciable results. Although the SVM classifier has several advantages that have been elucidated, it does have some limitations and uncertainties when it comes to building models for very large data sets. Moreover, the algorithm does not perform well for datasets where target classes are overlapping. It also underperforms in situations where the number of radiomic features for each data point exceeds the number of training data samples. The SVM will underperform in these situations.

Our study was a single institutional study with a quality-controlled central pathological laboratory and uniform radiology and radiomic review. One of the strengths of the study was that all the image delineation was verified by an experienced neuro-oncologist with 10 years of experience, blinded to the results of the molecular subgrouping. Being a tertiary cancer institute, it catered to a large and diverse pool of patients. The use of the single slice multiple sampling methods in this study not only helped in data augmentation but also prevented data loss. The main presumed weakness lies in the heterogeneity of MRI acquisition parameters in the study population and the fact that uniformity in image acquisition is necessary for radiomic analysis was acknowledged (29). Regardless of the heterogeneity in MR acquisition parameters, we were able to achieve a fair bit of accuracy, suggesting that this would consequently have a good implication if validated in a large cohort of patients in real-world clinical practice. Additionally, the current methodology of using internal cross-validation has the limitation of inflating the performance metrics. However, with a limited sample size, we thought that the internal 10-fold cross-validation would be the best strategy to utilize for model development. We are accruing more patients to evaluate the model on an external dataset, and this will be done in future studies.

In addition to radiomics features, our study did not include semantic features as those established by “The Visually AcceSAble Rembrandt Images” (VASARI) project could have potentially improved the performance of the model. Next, the study was limited by its small sample size with a skewed distribution of the various molecular subgroups. The relatively small sample size of our study also limited the use of deep learning algorithms, such as convolutional neural network (CNN) analysis, which requires a massive number of image datasets, which would not have been possible without the pooling of image data from multiple institutions, which in itself could have introduced a confounding factor of image heterogeneity, resulting in variability and generalization gaps in the predictive model. Although we did 10-fold internal cross-validation, the lack of an external validation cohort limits its robustness. These create future opportunities to incorporate clinical parameters and semantics features to complement the radiomic signatures to develop a more robust predictive model with better diagnostic metrics to classify the molecular subgroups of glioblastoma. The model developed in the current study is planned to be tested on an independent validation cohort and subsequently on a larger imaging dataset.

## Conclusion

The results of the study affirm that a texture feature-based radiomic model of multiparametric MR images can effectively classify molecular subgroups of GBM with an acceptable degree of accuracy using a machine learning approach. The proposed image-based radiomic approach provides an alternative non-invasive and efficient method to sub-classify the molecular subgroup and can aid in optimizing the adjuvant therapy of glioblastomas. Given that radiogenomics is rapidly evolving, machine learning approaches combined with clinical and radiological semantic (VASARI) features may show superior outcomes. The field of radiomics needs to be further researched to translate findings into an interpretable format for presurgical prediction of the molecular genotype of GBM.

## Data availability statement

The original contributions presented in the study are included in the article/**Supplementary Material**. Further inquiries can be directed to the corresponding author.

## Ethics statement

This study was reviewed and approved by the Institutional Ethics Committee,Tata Memorial Centre. Written informed consent for participation was not required for this study in accordance with the national legislation and the institutional requirements.

## Author contributions

PK, JSG, and AS contributed to the conception and design of the study. PK, ACS organized the database. PK and ACS performed the statistical analysis. PK, ACS, AS, AM, wrote the first draft of the manuscript. SE and AS wrote sections of the manuscript and did the immunohistochemistry and Sanger sequencing to classify IDH mutant vs wild type. All authors contributed to the manuscript revision, read and approved the submitted version. AS, JPA and JSG supervised the entire study.

## Conflict of interest

The authors declare that the research was conducted in the absence of any commercial or financial relationships that could be construed as a potential conflict of interest.

## Publisher’s note

All claims expressed in this article are solely those of the authors and do not necessarily represent those of their affiliated organizations, or those of the publisher, the editors and the reviewers. Any product that may be evaluated in this article, or claim that may be made by its manufacturer, is not guaranteed or endorsed by the publisher.

## Supplementary material

Supplementary Table 1 Shows the components of the Radiomic Quality score (RQS) with the total score of our study.

Supplementary Table 2 Shows the IBSI reporting of the study.
