# Supplementary material for: Predicting IDH subtype of grade 4 astrocytoma and glioblastoma from tumor radiomic patterns extracted from multiparametric magnetic resonance images using a machine learning approach
Source: Front Oncol. 2022 Sep 30;12:879376. doi: 10.3389/fonc.2022.879376 (PMC9585657; doi:10.3389/fonc.2022.879376)
Supplement: Supplementary Table 2 — Shows the IBSI reporting of the study. [file DataSheet_2.pdf]

Radiomics reporting guidelines and nomenclature as per IBSI (Image Biomarker Standardization Initiative)

Patient

Topic	Modality	Item	Description
Region of interest	*MRI*	1	Describe the region of interest that is being imaged:<br>*In our study, high-grade gliomas (grade 4 astrocytoma and glioblastoma) were imaged using multiparametric MRI, from which axial T2 and axial post-contrast T1 (T1C) weighted imaging sequences were utilized for this study.*
Patient preparation		2a	Describe specific instructions given to patients prior to image acquisition:<br>*The patient's serum creatinine/eGFR values are checked before contrast imaging.*
		2b	Describe administration of drugs to the patient prior to image acquisition:<br>*Not practiced routinely. In case of uncooperative or delirious patients, sedatives under expert anesthesia cover can be administered.*
		2c	Describe the use of specific equipment for patient comfort during scanning:<br>*Earplugs are provided for noise shielding. Additionally, warm blankets can be given as per patient requirements.*
Radioactive tracer	PET, SPECT	3a	Describe which radioactive tracer was administered to the patient, e.g. 18F-FDG:<br>*NA*
	PET, SPECT	3b	Describe the administration method:<br>*NA*
	PET, SPECT	3c	Describe the injected activity of the radioactive tracer at administration:<br>*NA*
	PET, SPECT	3d	Describe the uptake time prior to image acquisition:<br>*NA*
	PET, SPECT	3e	Describe how competing substance levels were controlled:<br>*NA*
Contrast agent		4a	Describe which contrast agent was administered to the patient:<br>*Gadolinium-based contrast agents (GBCA)*

		4b	Describe the administration method:<br>*Intravenous administration*
		4c	Describe the injected quantity of contrast agent:<br>*0.1mmol/ kg body weight of GBCA*
		4d	Describe the uptake time prior to image acquisition:<br>*NA*
		4e	Describe how competing substance levels were controlled:<br>*NA*
Comorbidities		5	Describe if the patients have comorbidities that affect imaging:<br>*None such amongst the patient population in our study.*

Acquisition

Topic	Modality	Item	Description
Acquisition protocol		6	Describe whether a standard imaging protocol was used, and where its description may be found:<br>*Prefixed brain tumor imaging protocol is used in the institute according to standardized recommendations.*
Scanner type		7	Describe the scanner type(s) and vendor(s) used in the study:<br>*MRI machines of 1.5T Philips Ingenia* ^{TM} *and 3T General Electric (GE)* ^{TM} *Signa were used in the study.*
Imaging modality		8	Clearly state the imaging modality that was used in the study:<br>*Multiparametric magnetic resonance imaging (MRI) was used in the study.*
Static/dynamic scans		9a	State if the scans were static or dynamic.<br>*Static scan*
	Dynamic scans	9b	Describe the acquisition time per time frame.<br>*NA*
	Dynamic scans	9c	Describe any temporal modeling technique that was used.<br>*NA*
Scanner calibration		10	Describe how and when the scanner was calibrated.<br>*In Philips Ingenia 1.5T, before every sequence, there is calibration for 5-6 seconds. In GE Signa 3T, calibration is done at the start of the scan. Pre-scan (preparatory scan) is done for 15-20 seconds at the beginning of each sequence.*

Patient instructions		11	Describe specific instructions given to the patient during acquisition:<br>*Instructions given to the patient include do not move, do not cough. If the patient feels the urge to move, he/she can press the call bell.*
Anatomical motion correction		12	Describe the method used to minimize the effect of anatomical motion:<br>*In GE Signa 3T, flow artifacts can be minimized by flow compensation. Propeller is a motion compensation imaging based on radiation k-space filling. No specific anatomical motion correction in Philips Ingenia 3T.*
Scan duration		13	Describe the duration of the complete scan or the time per bed position:<br>*The complete scan takes about 40-45 minutes in each of the scanners.*
Tube voltage	CT	14	Describe the peak kilo voltage output of the X-ray source.<br>*NA*
Tube current	CT	15	Describe the tube current in mA.<br>*NA*
Time-of-flight	PET	16	State if scanner time-of-flight capabilities are used during acquisition.<br>*NA*
RF coil	MRI	17	Describe what kind of RF coil is used for acquisition, incl. vendor.<br>*Philips Ingenia 1.5T uses ds Base, ds Posterior and Q-body coils.*<br>*GE HNS coil is used in GE Signa 3T.*
Scanning sequence	MRI	18a	Describe which scanning sequence was acquired.<br>*Standardized brain tumor imaging protocol encompasses multiparametric T1-weighted (T1W), T2-weighted (T2W), fluid-attenuated inversion recovery (FLAIR), diffusion-weighted imaging (DWI), T2*W gradient echo (GRE), and post-contrast T1W images (T1C).*<br>*Of which, axial T2 and axial post-contrast T1 weighted imaging were utilized for the study.*
	MRI	18b	Describe which sequence variant was acquired:<br>*No sequence variant was used, only spin echo sequences were used, T2-TSE (turbo spin echo) and T1-SE (spin echo).*
	MRI	18c	Describe which scan options apply to the current sequence:<br>*Flow compensation for vascular flow artifacts.*
Repetition time	MRI	19	Describe the time in ms between subsequent pulse sequences:

			In Philips Ingenia 1.5T, 5786ms for T2, and 571ms (500-600) for T1C.<br>*In GE Signa 3T, 4080ms for T2 and 400ms for T1+C.*
Echo time	MRI	20	Describe the echo time in ms:<br>*In Philips Ingenia 1.5T, 107ms for T2, and 22ms for T1+C. In GE Signa 3T, 85ms for T2 and minimum possible TE for T1+C.*
Echo train length	MRI	21	Describe the number of lines in k-space that are acquired per excitation pulse:<br>*In Philips Ingenia 1.5T, the turbo spin echo (TSE) is 16 for T2 and 10 for T1+C. In GE Signa 3T, it is 15 for T2 alone.*
Inversion time	MRI	22	Describe the time in ms between the middle of the inverting RF pulse to the middle of the excitation pulse: *NA, as not used in our study.*
Flip angle	MRI	23	Describe the flip angle produced by the RF pulses:<br>*The flip angle is at 90 degrees for each of the spin echo sequences in both the scanners.*
Acquisition type	MRI	24	Describe the acquisition type of the MRI scan:<br>*2D axial T2 and post-contrast T1 weighted imaging.*
k-space traversal	MRI	25	Describe the acquisition trajectory of the k-space:<br>*The points in the k-space are acquired through frequency encoding and successive phase encoding steps. Once the entire matrix is filled in, the inverse Fourier transform decodes the frequency information from the actual image. Each line of k-space is a separate phase encoding step. No anatomical motion correction in Philips Ingenia 1.5T.*
Number of averages/ excitations	MRI	26	Describe the number of times each point in k-space is sampled:<br>*NEX 2 in Philips Ingenia 1.5T and NEX 1 in GE Signa 3T.*<br><br>*NEX: Number of excitations*
Magnetic field strength	MRI	27	Describe the nominal strength of the MR magnetic field:<br>*1.5Tesla for Philips Ingenia™ and 3Tesla for GE Signa™.*

Reconstruction

Topic	Modality	Item	Description
In-plane resolution		28	Describe the distance between pixels, or alternatively the field of view and matrix size.<br>*In GE Signa 3T*<br>*Axial T2: FOV 24cm, Matrix size 320 x 224, NEX: 1*<br>*Axial T1+C: FOV 24cm, Matrix size 320 x 190, NEX: 1*

			In Philips Ingenia 1.5T<br>*Axial T2: FOV 23 (AP) 18.5 (RL), Matrix size 448 x 304, NEX: 2*<br>*Axial T1+C: FOV 23 (AP) 18.5 (RL), Matrix size 232 x 104, NEX: 2*<br><br>*FOV: Field-of-view*<br>*AP: Anteroposterior*<br>*RL: Right left*
Image slice thickness		29	Describe the slice thickness:<br>*5mm in GE Signa 3T, 5mm in Philips Ingenia 1.5T*
Image slice spacing		30	Describe the distance between image slices:<br>*1.5mm in GE Signa 3T, 1 mm in Philips Ingenia 1.5T*
Convolution kernel	CT	31a	Describe the convolution kernel used to reconstruct the image.: *NA*
	CT	31b	Describe settings pertaining to iterative reconstruction algorithms: *NA*
Exposure	CT	31c	Describe the exposure (in mAs) in slices containing the region of interest. *NA*
Reconstruction method	PET	32a	Describe which reconstruction method was used, e.g. 3D OSEM. *NA*
	PET	32b	Describe the number of iterations for iterative reconstruction. *NA*
	PET	32c	Describe the number of subsets for iterative reconstruction. *NA*
Point spread function modeling	PET	33	Describe if and how point-spread function modeling was performed. *NA*
Image corrections	PET	34a	Describe if and how attenuation correction was performed. *NA*
	PET	34b	Describe if and how other forms of correction were performed, e.g. scatter correction, randoms correction, dead time correction, etc. *NA*
Reconstruction method	MRI	35a	Describe the reconstruction method used to reconstruct the image from the k-space information:<br>*The MR signals derived from each phase encoding step are stored in a raw data matrix, known as k-space. A two-dimensional Fourier transformation of this matrix results in the reconstruction of the image.*<br>*In Philips 1.5T, CS sense is used in T2W imaging. This is not applicable in T1+C sequences.*

	MRI	35b	Describe any artifact suppression methods used during reconstruction to suppress artifacts due to undersampling of k-space: *Increasing the matrix size and NEX can be used to increase the k-space filling. Also, increase the fold-over suppression and change the phase encoding direction.*
Diffusion-weigh ted imaging	DWI-MRI	36	Describe the b-values used for diffusion-weigh ting. *We did not use DWI MRI for our radiomics study*

Image registration

Topic	Modality	Item	Description
Registration method		37	Describe the method used to register multi-modality imaging: *No registration is needed for these sequences.*

Image processing

Data conversion

Topic	Modality	Item	Description
SUV normalization	PET	38	Describe which standardized uptake value (SUV) normalization method is used. *NA*
ADC computation	DWI-MRI	39	Describe how apparent diffusion coefficient (ADC) values were calculated. *NA, as this sequence was not utilized in our study.*
Other data conversions		40	Describe any other conversions that are performed to generate: *Perfusion maps are used in imaging high-grade gliomas, however, this was not included in the study*.

Post-acquisition processing

Topic	Modality	Item	Description
Anti-aliasing		41	Describe the method used to deal with anti-aliasing when down-sampling during interpolation. *NA*
Noise suppression		42	Describe methods used to suppress image noise.

			TexRAD uses Laplacian of Gaussian bandpass filters to remove noise (Gaussian filter) and to enhance edges (Laplacian filter).
Post-reconstruction smoothing filter	PET	43	Describe the width of the Gaussian filter (FWHM) to spatially smooth intensities. *NA*
Skull stripping	MRI (brain)	44	Describe the method used to perform skull stripping. *NA*
Non-uniformity correction	MRI	45	Describe the method and settings used to perform non-uniformity correction. *Non-uniformity corrections not done.*
Intensity normalization		46	Describe the method and settings used to normalize intensity distributions within a patient or patient cohort. *Not done. Filtration algorithms are used to reduce the noise.*
Other post-acquisition processing methods		47	Describe any other methods that were used to process the image and are not mentioned separately in this list. *NA*

Segmentation

Topic	Modality	Item	Description
Segmentation method		48a	Describe how regions of interest were segmented, e.g. manually. *The segmentation was done manually.*
		48b	Describe the number of experts, their expertise, and consensus strategies for manual delineation. *The initial segmentation was done by a radiologist having 2 years of experience in neuroradiology. The ROI contours and segmentation were separately verified by a neuro-oncologist having 10 years of experience and a neuroradiologist having 10 years of experience. The segmentation was verified by them individually and any discrepancy was resolved by a consensus. For the purpose of analysis, the final contours as verified by the neuroradiologist were taken into consideration.*
		48c	Describe methods and settings used for semi-automatic and fully automatic segmentation. NA
		48d	Describe which image was used to define segmentation in the case of multi-modality imaging.

			Axial T2 and axial post-contrast T1 (T1C) weighted imaging was used.
Conversion to mask		49	Describe the method used to convert polygonal or mesh-based segmentations to a voxel-based mask. *NA*

Image interpolation

Topic	Modality	Item	Description
Interpolation method		50a	Describe which interpolation algorithm was used to interpolate the image. *We did not use any interpolation algorithm. The ROI in all the slices was manually segmented*
		50b	Describe how the position of the interpolation grid was defined, e.g. align by the center. *NA*
		50c	Describe how the dimensions of the interpolation grid were defined, e.g. rounded to the nearest integer. *NA*
		50d	Describe how extrapolation beyond the original image was handled. *NA*
Voxel dimensions		51	Describe the size of the interpolated voxels. *NA*
Intensity rounding	CT	52	Describe how fractional Hounsfield Units are rounded to integer values after interpolation. *NA*

ROI interpolation

Topic	Modality	Item	Description
Interpolation method		53	Describe which interpolation algorithm was used to interpolate the region of interest mask. *We did not use any interpolation method.*
Partially masked voxels		54	Describe how partially masked voxels after interpolation are handled. *NA*

Re-segmentation

Topic	Modality	Item	Description

Re-segmentation methods		55	Describe which methods and settings are used to re-segment the ROI intensity mask. *Resegmentation was not done*

Discretization

Topic	Modality	Item	Description
Discretization method		56a	Describe the method used to discretize image intensities. *We did not discretize image intensities on the radiomic software*
		56b	Describe the number of bins (FBN) or the bin size (FBS) used for discretization. *NA*
		56c	Describe the lowest intensity in the first bin for FBS discretization. *NA*

Image transformation

Topic	Modality	Item	Description
Image filter		57	Describe the methods and settings used to filter images, *Laplacian-of-Gaussian filters*

Image biomarker computation

Topic	Modality	Item	Description
Biomarker set		58	Describe which set of image biomarkers is computed and refer to their definitions or provide these. *Please find the definitions below- Table I*
IBSI compliance		59	State if the software used to extract the set of image biomarkers is compliant with the IBSI benchmarks. *Partially compliant*
Robustness		60	Describe how the robustness of the image biomarkers was assessed, e.g. test-retest analysis. *1. LASSO Regression was used to select robust features for the endpoint of interest. LASSO regression removes redundant features and keeps only those radiomic features that are highly associated with the endpoint of interest.*

			2. The ML classifier of SVM used a kernel-based 10-fold internal cross-validation strategy to increase the robustness of the obtained results.
Software availability		61	Describe which software and version were used to compute image biomarkers. *TexRAD Research+ Version 3.10, TexRAD Ltd, Cambridge, UK*

Image biomarker computation - texture parameters

Topic	Modality	Item	Description
Texture matrix aggregation		62	Define how texture-matrix-based biomarkers were computed from underlying texture matrices. *GLCM parameters – features are computed from each 2D directional matrix and averaged over 2D directions and slices (IBSI-BTW3).*
Distance weighting		63	Define how CM, RLM, NGTDM, and NGLDM weight distances, e.g. no weighting. *No weighting was done.*
CM symmetry		64	Define whether symmetric or asymmetric co-occurrence matrices were computed. *Symmetric matrices were computed.*
CM distance		65	Define the (Chebyshev) distance at which co-occurrence of intensities is determined, e.g. 1. *Chebyshev distance – 1 (GLCM1) and 4 (GLCM4) was used*.
SZM linkage distance		66	Define the distance and distance norm for which voxels with the same intensity are considered to belong to the same zone for the purpose of constructing an SZM, e.g. Chebyshev distance of 1. *NA*
DZM linkage distance		67	Define the distance and distance norm for which voxels with the same intensity are considered to belong to the same zone for the purpose of constructing a DZM, e.g. Chebyshev distance of 1. *NA*
DZM zone distance norm		68	Define the distance norm for determining the distance of zones to the border of the ROI, e.g. Manhattan distance. *NA*
NGTDM distance		69	Define the neighbourhood distance and distance norm for the NGTDM, e.g. Chebyshev distance of 1. *NA*
NGLDM distance		70	Define the neighbourhood distance and distance norm for the NGLDM, e.g. Chebyshev distance of 1. *NA*

NGLDM coarseness		71	Define the coarseness parameter for the NGLDM, e.g. 0. *NA*

Machine learning and radiomics analysis

Topic	Modality	Item	Description
Diagnostic and prognostic modeling		72	See the TRIPOD guidelines for reporting on diagnostic and prognostic modeling. *The TRIPOD guidelines are not applicable in the study as we are investigating radiomics for molecular subclassification of gliomas.*
Comparison with known factors		73	Describe where the performance of radiomics models is compared with known (clinical) factors. *Not applicable.*
Multicollinearity		74	Describe where the multicollinearity between image biomarkers in the signature is assessed. *Multicollinearity affects linear models. The non-linear SVM model used in this study is not greatly impacted by multicollinearity.*
Model availability		75	Describe where radiomics models with the necessary pre-processing information may be found. *The radiomics modeling was done using a support vector machine tool on MATLAB software.*
Data availability		76	Describe where imaging data and relevant meta-data used in the study may be found. *The anonymized imaging data and the relevant metadata are stored in a secure database with access only to the Principal investigator. The data can be made available only after the institutional ethics committee allows the PI.*

Table I:

1st Order Statistics	***Description***
Mean	*The average value of the pixels within the region of interest.*
Standard deviation	*Variation or dispersion from the mean.*
Skewness	*Asymmetry of the histogram*
Kurtosis	*Peakedness/ Flatness of the histogram*
Mean of positive pixels (MPP)	*Considers only pixels greater than 0*
Entropy	*Irregularity of gray-level distribution*
Uniformity	*Uniformity of gray-level distribution.*
2nd Order Statistics	***Description***
Entropy	*Measures the degree of disorder among pixels in the image.*

Sum entropy	*Measures the disorder related to the gray level sum distribution of the image.*
Difference entropy	*Measures the disorder related to the gray level difference distribution of the image.*
Energy (or Angular Second Moment)	*The measure of homogeneous patterns in the image.*
Dissimilarity	*Measurement of how different each element of gray-level voxel pair is.*
Correlation	*Measures linear dependency of gray level values to their respective voxels in the GLCM*
Autocorrelation	*The measure of the magnitude of the fineness and coarseness of texture, a higher value indicates a texture with more pairs with high gray levels.*
Contrast	*Signifies the amount of local gray level disparity in an image; a higher value indicates the presence of edges, noise, or wrinkled textures in the image.*
Homogeneity (inverse difference moment)	*The smoothness (homogeneity) of the gray level distribution of the image.*
Variance (sum of squares)	*The dispersion of the gray level distribution.*
Sum average	*Measures the mean of the gray level sum distribution of the image.*
Sum variance	*Measures the dispersion (concerning the mean) of the gray level sum distribution of the image.*
Difference variance	*Measures the dispersion (concerning the mean) of the gray level difference distribution of the image.*
Joint Average	*Return the mean gray level intensity of the distribution.*
Cluster Prominence	*The measure of the skewness and asymmetry of the GLCM.*
Cluster Shade	*The measure of the skewness and uniformity of the GLCM.*
Cluster Tendency	*The measure of groupings of voxels with similar gray-level values.*
Difference Average	*Measures the relationship between occurrences of pairs with similar intensity values and occurrences of pairs with differing intensity values.*
Difference Entropy	*The measure of the randomness/variability in neighborhood intensity value differences.*
Difference Variance	*The measure of heterogeneity that places higher weights on differing intensity level pairs that deviate more from the mean.*
Inverse Difference Moment (IDM)	*The measure of the local homogeneity of an image.*
Inverse Difference Moment Normalized (IDMN)	*Also, a measure of the local homogeneity of an image.*
